# Supplementary material for: Seroepidemiological investigation of Crimean Congo hemorrhagic fever virus in livestock in Uganda, 2017
Source: PLoS One. 2023 Nov 9;18(11):e0288587. doi: 10.1371/journal.pone.0288587 (PMC10635543; doi:10.1371/journal.pone.0288587)
Supplement: Attachment — Submitted filename: Response to reviewers.docx [file pone.0288587.s002.docx]

Reviewers' comments:

 Abstract:

1. In this sentence “CCHFV seropositivity appeared to be generally higher in northern districts of the country, though spatial trends among sampled districts were not obvious”, I suggest replacing ‘obvious’ to ‘examined’.

**Thank you for the feedback. The recommended correction has been made**

2. Make sure that decimals all along are the same, either 1 or 2. For example the total of the percentages of seropositive animals for all species does not add 100%. Please check this all along.

Methods:

1. “Herds were selected for sampling to be followed prospectively” – Not sure what this means, adding the word prospectively to the methods when this is a survey is confusing. Please clarify or remove.

2. “Assuming an average of 15 animals per herd, we 118 expected to sample 156 herds, distributed evenly throughout the 27 districts selected for sampling”- Ok, but it would be good to add how you selected the herds within each district and the 15 animals within each herd (when the herd had more than 15 animals). Add one sentence if each one of these steps was random or convenience sampling.

**Clusters of herds were purposively selected based on specific criteria, including those with a high tick burden, high animal population and cooperative animal owners, those located in dry and wet areas, and those situated near international borders. Once the clusters were identified, herds with 15 or fewer animals were entirely sampled, while herds with more than 15 animals were sampled using proportional size sampling, where only 25% of the herd was selected for sampling. Individual animals were conveniently chosen and restrained in a crush, and blood samples were collected until the 25% achieved. This has been added in the manuscript.**

Results:

1. In table 1 please check that all the total of the animals for each variable is 3181. Some are not at the moment (e.g. Sex). Suggest to double check too in table 2 and 3, just in case.

**Thanks for this. We have checked the tables for clarity**

2. “Holding all other variables in the model constant, the associations between CCHF seropositivity and animal breed and elevation were not statistically significant based on a 95% confidence limit, however, the estimated odds of seropositivity were lower among cross and exotic breeds compared to indigenous breeds, and the estimated odds of seropositivity among animals at lower elevations was higher than that among animals at higher elevations (Table 3)” – Don’t agree with what you say after 'however' because clearly you have your odds ratio indicating that based on your data there is no association. Suggesting otherwise might be misleading, maybe if you’d like to leave it better for a point of discussion.

**Thanks for this observation. We have removed the last component**

Discussion

1. “However, Balinandi et al (2021) also performed serology on cattle samples using 278 a commercial CCHF serological testing kit, the ID screen CCHF double antigen multi-species 279 (IDVet), and found seropositivity of 75% in cattle (Balinandi, von Brömssen, et al., 2021; Sas et 280 al., 2018), thus suggesting some commercial assays may overestimate the true livestock 281 seroprevalence of CCHFV” – Still not sure if you can say this… not sure unless you’re sure it is the same area, same population and timing or if you have any evidence to support the claim that commercial tests overestimate CCHFv seroprevalence.

**After careful consideration, we have concluded and acknowledged that we lack sufficient evidence to assert an overestimation of seropositivity by commercial ELISAs. As a result, we have made appropriate revisions to the statement, which now states:**

**Balinandi et al. (2021) performed serology tests on cattle samples in three of the studied districts using the ID screen CCHF double antigen multi-species (IDVet), a commercial CCHF serological testing kit. Their findings revealed a seropositivity rate of 75% in cattle. This suggests that the selection of ELISA assay could potentially result in variations in seropositivity rates(Balinandi, von Brömssen, et al., 2021; Sas et al., 2018).**

2. “We also found that the odds of seropositivity were higher among 315 animals sampled at lower elevations. Seroprevalence was also higher in animals with reported 316 stillbirth and abortion” – Agree but some of these aspects didn’t go to the multivariable model or didn’t come up as significant when all variables considered, for example elevation. I suggest that you make this distinction because sounds like you’d really like elevation to be one of the variables associated, but your risk model does not support that.

**We appreciate your astute observation, and as a result, we have revised the paragraph to provide an explanation for why we were unable to include these variables in the multivariable model due to missing data**.

3. “However, as CCHF is not known to be symptomatic in animals, there is a 317 need to investigate further the impact of the CCHFV infection on animal production in terms of 318 reducing herd size and milk production. However, the interpretation and association of CCHFV 319 seropositivity with stillbirth and abortion could be confounding since the same animal populations 320 in Uganda are susceptible to other diseases that cause abortions such as brucellosis and Rift Valley 321 fever virus” – Double use of however, maybe rephrase.

**This paragraph has been rephrased.**

4. “However, the interpretation and association of CCHFV 319 seropositivity with stillbirth and abortion could be confounding since the same animal populations 320 in Uganda are susceptible to other diseases that cause abortions such as brucellosis and Rift Valley 321 fever virus” – True, also the fact that this is a cross sectional study makes it impossible to test causation, only association.
**This is correct observation.**
5. “However, a stratified analysis limited to adult animals produced an odds ratio 336 approximately equal to that in the full analysis for female animals compared to male animals, 337 suggesting that age is not the primary explanation for the difference in seroprevalence between 338 male and female animals.” – Seems like adding results here, beware how you present this as it was not in your results.

**Thanks for this observation, this section has been edited out.**
